# A sustainable public health framework for PCOS management in low- and middle-income countries: a narrative review

**DOI:** 10.3389/frph.2025.1627670

**Published:** 2025-09-18

**Authors:** Sudharsan Vasudevan, Rohit Gautam, Pratibha Maan, Amit Arora, Ashraf Ganie, Puthiyaveettil Khadar Jabbar, Taruna Arora

**Affiliations:** ^1^ Division of RCN, Indian Council of Medical Research, New Delhi, India; ^2^ Sir Ganga Ram Hospital, New Delhi, India; ^3^ Sher-i-Kashmir Institute of Medical Sciences, SKIMS, Srinagar, Jammu & Kashmir, India; ^4^ Government Medical College, Thiruvananthapuram, Kerala, India

**Keywords:** PCOS, LMICs, public health, diagnosis, treatment, SDG goals

## Abstract

**Background:**

Polycystic Ovary Syndrome is an endocrine disorder that affects reproductive, metabolic, and mental health. In LMICs, PCOS management is hindered by late diagnosis, lack of awareness, and high treatment costs which leads to long-term complications.

**Objective:**

The aim of the review is to document the challenges in PCOS diagnosis and management in LMICs and provide public health solution to overcome these barriers in accordance with SDG goals.

**Methods:**

A narrative review synthesizing existing literature on PCOS epidemiology, barriers to diagnosis and treatment, and potential solutions relevant to LMICs.

**Results:**

Key challenges include lack of uniformity in diagnosis and treatment, lack of trained HR and equipment. High cost of care, stigma and fragmented health care.

**Outcomes/proposed solutions:**

Develop national PCOS guidelines, bring the management of PCOS under the reproductive health program, shift some of the tasks to primary health workers, like generating awareness and screening for symptoms. Invest in research to find public health solutions.

**Conclusion:**

Addressing PCOS in LMICs requires a multi-sectoral public health approach, including prevention, early detection, and affordable care. Strengthening healthcare systems through policy reforms and community-based interventions can improve outcomes for affected women.

## 1 Background

Polycystic Ovary Syndrome (PCOS) is one of the most common disorders among reproductive-age women worldwide. The Rotterdam criteria defines it as a combination of features like hyperandrogenism, ovulatory dysfunction, and polycystic ovarian morphology (1). PCOS is characterized by a heterogeneous range of clinical features, including oligomenorrhea, anovulation and infertility on the one end, and metabolic disorders such as hyperinsulinemia, insulin resistance and obesity on the other (2). These symptoms often overlap with other health conditions, complicating the diagnosis and management of the disorder. Globally, the prevalence of PCOS is estimated to range between 6% and 20%, depending on the diagnostic criteria and population studied. In India, however, the prevalence is alarmingly high, with reports suggesting rates as high as 36% among women of reproductive age (2). This disparity is often attributed to rapid urbanization, increasing rates of obesity, and lifestyle changes among Indian women. The higher prevalence of PCOS in urban settings compared to rural areas further underscores the role of environmental and lifestyle factors in its pathogenesis (3). The impact of PCOS extends beyond reproductive health, significantly affecting metabolic and psychological well-being. Women with PCOS are at a higher risk of developing cardiovascular diseases and non-alcoholic fatty liver disease as well (4). In addition, the psychological burden of PCOS, including increased rates of anxiety, depression, and body image issues, often remains underdiagnosed and untreated (5). These complications highlight the need for holistic approaches to the diagnosis and management of PCOS, particularly in low-resource settings where healthcare systems are already overburdened.

The complexities involved in the diagnosis and management of PCOS are more pronounced in low—resource settings than in developed ones. Low and middle income countries (LMICs) are part of a classification system developed by World bank that classifies based on gross national income capita, where healthcare resources are limited (6). These countries have issues like poor developed healthcare, insufficiently trained medical personal, higher costs of tests and drugs because of reduced availability (7). These include problems like lack of recognition of the condition by women and health workers as well as the socio-economic strains and the underdeveloped health care system (8). It is common to see a late diagnosis of PCOS since many women go to the doctor only when they have infertility or other advanced complications of PCOS (9). A study in Bangladesh reiterates the problem of delayed diagnosis in PCOS lack of awareness about PCOS among women (10). Health disparities between urban and rural areas also cause the problem to become more complex. While metropolis may offer women professionals and advanced testing equipment, rural women depend on primary healthcare providers, who may not have the necessary training or resources to diagnose and treat PCOS effectively (9). A study from Nigeria discusses issues with awareness, and poor health infrastructure affecting PCOS care (11). This disparity is further exacerbated by social taboos on discussions concerning menstrual and reproductive health, and thus, the society hinders the opportunity for quick medical assistance. One of the socioeconomic aspects also in fact that the cost involved in acquiring these diagnostic tests is one of the major barriers to accessing medical care. The costs of diagnostic tests like hormone assays and ultrasound imaging that are done on the ovary, and which are not covered by insurance, make the tests unaffordable to many in low-income situations (12–14). Lack of integrated care also affects PCOS management, due to which only selective management of PCOS manifestations gets treated due to their obvious presentation, while long term complications get neglected (13, 15, 16).

Efforts to address PCOS in these settings require a comprehensive approach that includes preventive measures, early diagnosis, and community-based interventions (1, 8). Community-based healthcare models have shown promise in bridging the gap between urban and rural healthcare disparities (17). Telemedicine platforms also offer a viable solution for connecting women in remote areas with specialists, reducing the need for travel and associated costs (18). The 2015 Sustainable development goals (SDG) 3, 5, 10 talk about achieving health for all, gender equality and reducing barriers to health across for all groups by 2030, PCOS being an illness that disproportionately affects a woman of all ages across the world, needs to be tackled head on to achieve those targets (7, 8). This review tries to bridge the gaps of clinician led approach to tacking this illness by suggesting public health and policy solutions by highlighting varies models and approaches that have been used across the globe. The aim of the review is to document the challenges in PCOS diagnosis and management in LMICs and provide public health solution to overcome these barriers in accordance with SDG goals.

## 2 Challenges to PCOS management in LMIC

**Figure 1:**
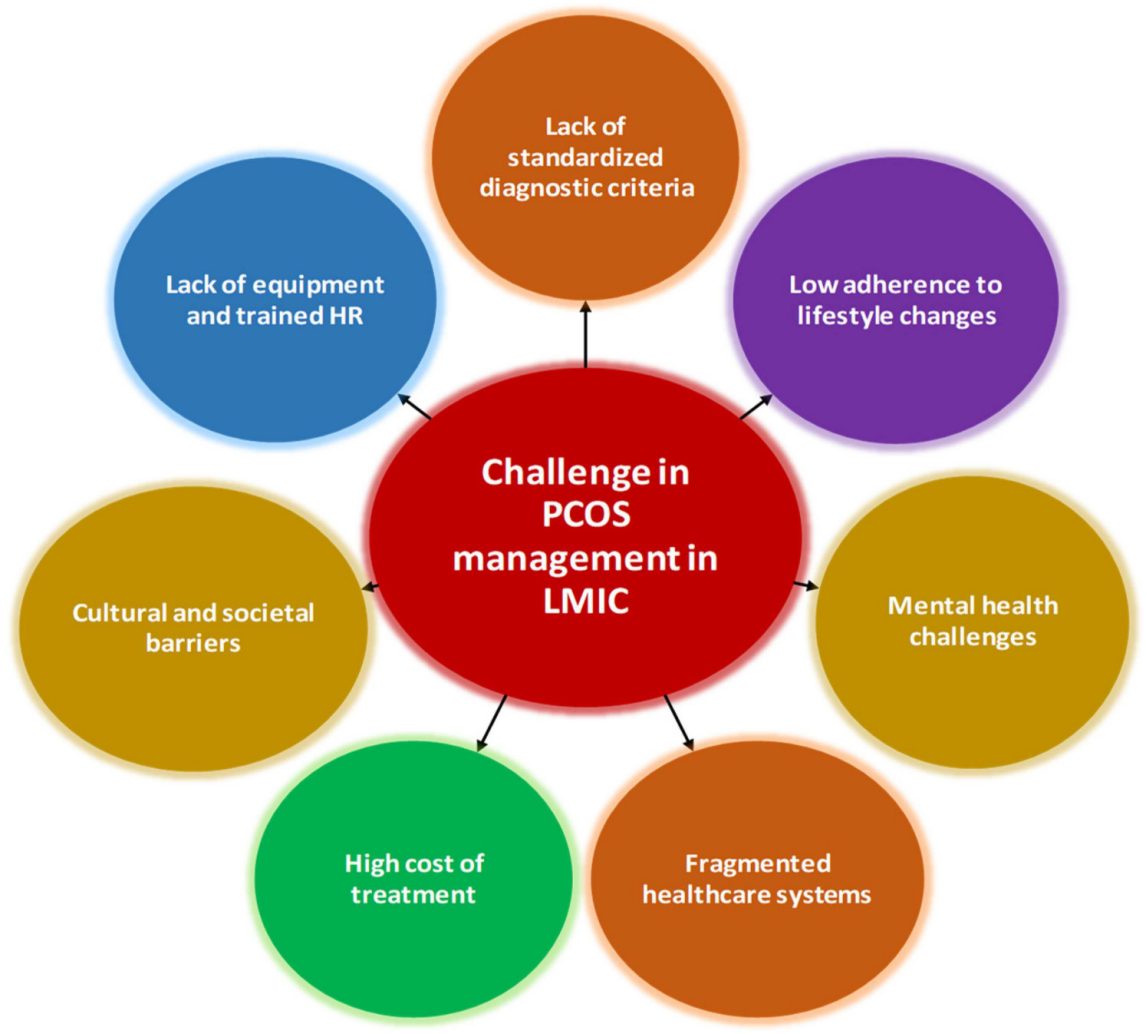
Bottlenecks for PCOS management in LMICs.

This section describes the various bottle necks that prevent PCOS women of LMICs from getting holistic care, which includes challenges in diagnosis and treatment (Figure 1). Addressing these challenges requires a multifaceted approach. Standardizing diagnostic criteria, improving access to diagnostic tools, training healthcare providers, and integrating multidisciplinary care are critical steps (19, 20).

### 2.1 Challenges in diagnosis

#### 2.1.1 Lack of standardised diagnostic criteria

The absence of uniform diagnostic criteria for PCOS the world is one of the barriers for PCOS diagnosis. The 3 most common guidelines are the 2003 Rotterdam Consensus and the criteria of the National Institutes of Health (NIH) and the Androgen Excess and PCOS Society (AE-PCOS) (21).

Rotterdam criteria: Claims that two of the following three features should be hyperandrogenism (HA), oligo/anovulation (OA), and polycystic ovarian morphology (PCOM) on ultrasound for a woman to full fill the PCOS classification, while the absence of the remaining feature is being compensated by two other features (22).

NIH: Specifies that the diagnosis should include hyperandrogenism and oligo/anovulation but exclude the ultrasound findings (23).

AE-PCOS Criteria: States that women should have hyperandrogenism and at least one other feature to be diagnosed with PCOS (24).

Due to the variability of the disease, the symptoms of PCOS it is often a matter of controversy among medical practitioners and sometimes the diagnosis is inconsistent especially in primary care settings. Women are often treated without adequate diagnosis which leads to inappropriate treatment. Furthermore, the condition can worsen (1).

The diagnosis of PCOS is further complicated by varying phenotypic presentations such as phenotype-A which includes all the three criteria for diagnosis, phenotype-B includes hyper androgenic and oligo anovulatory features (HA + OA), phenotype-C has features of hyper androgenism and polycystic ovaries (HA + PCOM), and phenotype-D (OD + PCOM). Phenotype-A is more common in subjects identified in clinical populations, whereas phenotype-C is more common in unselected populations (22, 25).

In low-resource settings, a simpler diagnostic method based on clinical features like oligomenorrhoea and hirsutism might be a practical alternative to the full Rotterdam criteria. This is particularly true when hormonal tests or pelvic ultrasounds are not possible. These models have been suggested as useful tools for early detection and referral from primary care (24, 26).

#### 2.1.2 Lack of equipment and trained human resources (HR)

In rural and poor settings, access to diagnostic tools such as high-resolution ultrasound and hormonal assays is limited and knowledge on how to diagnose and screen this disorder is limited (27, 28). Polycystic ovarian morphology, a key diagnostic criterion in the Rotterdam guidelines, cannot be assessed without ultrasound imaging, trained health care provides who can perform ultrasounds are limited in primary care set up. Hormonal assays for measuring androgen levels are equally challenging to access due lack of specialised technicians in rural healthcare facilities (29). As a result, diagnosis in these settings often relies on clinical evaluations, which are insufficiently sensitive (30).

Symptoms related to puberty like irregular periods, acne, and hirsutism may be misinterpreted as normal development or other health issues. This can lead to delay in the right diagnosis and effective treatment (30). Therefore, it is more important to recognize Polycystic Ovary Syndrome (PCOS) at an early stage among adolescents (31, 32). On the other hand, a study in India showed that many adolescent PCOS cases were not diagnosed due to the failure of healthcare providers to identify the vague presentation of the syndrome (2).

PCOS also has symptoms that are common with other medical conditions entitled, thyroid diseases, hyperprolactinemia, and adrenal hyperplasia (33, 34). This shared symptom presentation further hinders the diagnosis process (4).

#### 2.1.3 Cultural and societal barriers

In many cultures, menstruation and infertility are stigmatized topics**,** discouraging women from seeking medical attention (31, 33). The stigma surrounding menstrual irregularities leads to delays in diagnosis and treatment, particularly in conservative societies (33). Additionally, a lack of awareness about PCOS among women themselves compounds the problem, as symptoms are often normalized or ignored (8). Studies from Myanmar, Nepal and Lebanon talk about disaster scenarios in the region where women hardly have access to menstrual education, or menstrual products (35, 36).

### 2.2 Challenges in PCOS treatment

#### 2.2.1 High cost of treatment

PCOS management often involves a combination of pharmacological and non-pharmacological approaches for several years. Medications like oral contraceptives, metformin, and clomiphene citrate are frequently prescribed in tandem (37). Long term complications of PCOS also require muti disciplinary specialised care, these services are expensive and often unavailable in low-income settings. Lifestyle modification programs, a cornerstone of PCOS treatment, also require resources such as dieticians and fitness professionals, which are inaccessible to many women (38). Many LMICs lack universal healthcare, leading to them having to send from their own pockets, this leads to catastrophic expenditure (39) (Table 1).

**Table 1:** Key diagnostic and treatment challenges of PCOS.

Challenge	Description	Evidence and references
Lack of standardized diagnostic criteria	Inconsistent criteria (Rotterdam, NIH, AE-PCOS Society) cause diagnostic variability. Phenotypic differences complicate diagnosis further.	Variability of PCOS symptoms and diagnostic criteria leads to inconsistent diagnoses (23, 40).
Lack of equipment and trained human resources	Limited access to ultrasound, hormonal assays, and trained specialists in rural settings results in under diagnosis.	Insufficient access to diagnostic tools and clinical knowledge in primary care settings (25, 30).
Cultural and societal barriers	Menstruation and infertility stigma discourage women from seeking care, leading to delays in diagnosis and treatment.	Cultural stigmas surrounding menstrual health and infertility in conservative societies delay care-seeking (31, 33).
High cost of treatment	Long-term PCOS management requires medications and lifestyle modifications, which are often inaccessible due to high costs.	Out-of-pocket expenses in LMICs can lead to catastrophic expenditure (7, 39).
Fragmented healthcare systems	Lack of multidisciplinary care results in symptom-based treatments, primarily for infertility, with long-term complications overlooked.	Short-term treatment goals dominate care due to fragmented systems and lack of follow-up (41, 42).
Mental health challenges	High rates of anxiety, depression, and body image issues are prevalent but rarely addressed in PCOS treatment.	Mental health services are often absent or stigmatized in PCOS management, especially in low-resource settings (43, 44).
Low adherence to lifestyle changes	Socioeconomic barriers, cultural factors, and lack of structured programs hinder adherence to lifestyle interventions.	Women in rural areas lack safe spaces for physical activity and access to nutritious food (45, 46).

Metformin is one of the most affordable medications for diabetes, PCOS and other metabolic syndrome (47). It significantly reduces insulin resistance and high androgen levels while improving ovulation (48). Its availability as a generic drug and low monitoring needs makes it a great choice for health systems in low to middle-income countries.

#### 2.2.2 Fragmented healthcare systems

The treatment of PCOS requires a team of gynaecologists, endocrinologists, dietitians, and mental health professionals work together, but many a case, these are not available in rural as well as in other low resource settings, the specialists if present tend to work on only one aspect of the syndrome and do not take sufficient care to council with rest of the other specialists to provide comprehensive care (41). Due to which a symptom-based approach to treatment is followed, catering predominantly to patients with problems like infertility, which is more commonly known and hence the collaborative approach to treatment is not followed (49).

PCOS is associated with long-term issues such as type 2 diabetes, cardiovascular diseases, and endometrial cancer. Despite this, treatment in many settings remains focused on short-term goals like regulating menstrual cycles or inducing ovulation. Long-term complications are often overlooked, particularly in low-resource settings where regular follow-up and preventive care are unavailable (1).

#### 2.2.3 Mental health challenges

Anxiety, depression, and body image issues are prevalent among women with PCOS, with studies indicating significantly higher rates compared to the general population (50). Despite this, mental health is rarely addressed in PCOS treatment plans, particularly in low-resource settings where psychological services are either unavailable or stigmatized (43).

#### 2.2.4 Low adherence to lifestyle changes

Lifestyle modification is a cornerstone of PCOS management, yet adherence remains low due to socioeconomic barriers, lack of support, and cultural factors (45). For instance, women in rural areas may not have access to safe spaces for physical activity or affordable, nutritious food. Additionally, the absence of structured lifestyle intervention programs in low-resource settings further limits their effectiveness.

Summary of the challenges in PCOS diagnosis and management, in LMIC context, in Nepal, sociocultural norms often discourage adolescent girls from seeking reproductive health care, compounding delays in PCOS diagnosis (35). In Ethiopia, PCOS is underdiagnosed due to a lack of trained gynaecologists outside urban centers (23). In Brazil and Mexico, PCOS awareness is significantly lower in rural communities compared to urban counterparts, leading to delayed care and higher disease burden (9). These insights have been incorporated to demonstrate how public health responses must be tailored to reflect each country's epidemiological profile, service availability, and cultural context.

## 3 Public health solutions

**Figure 2:**
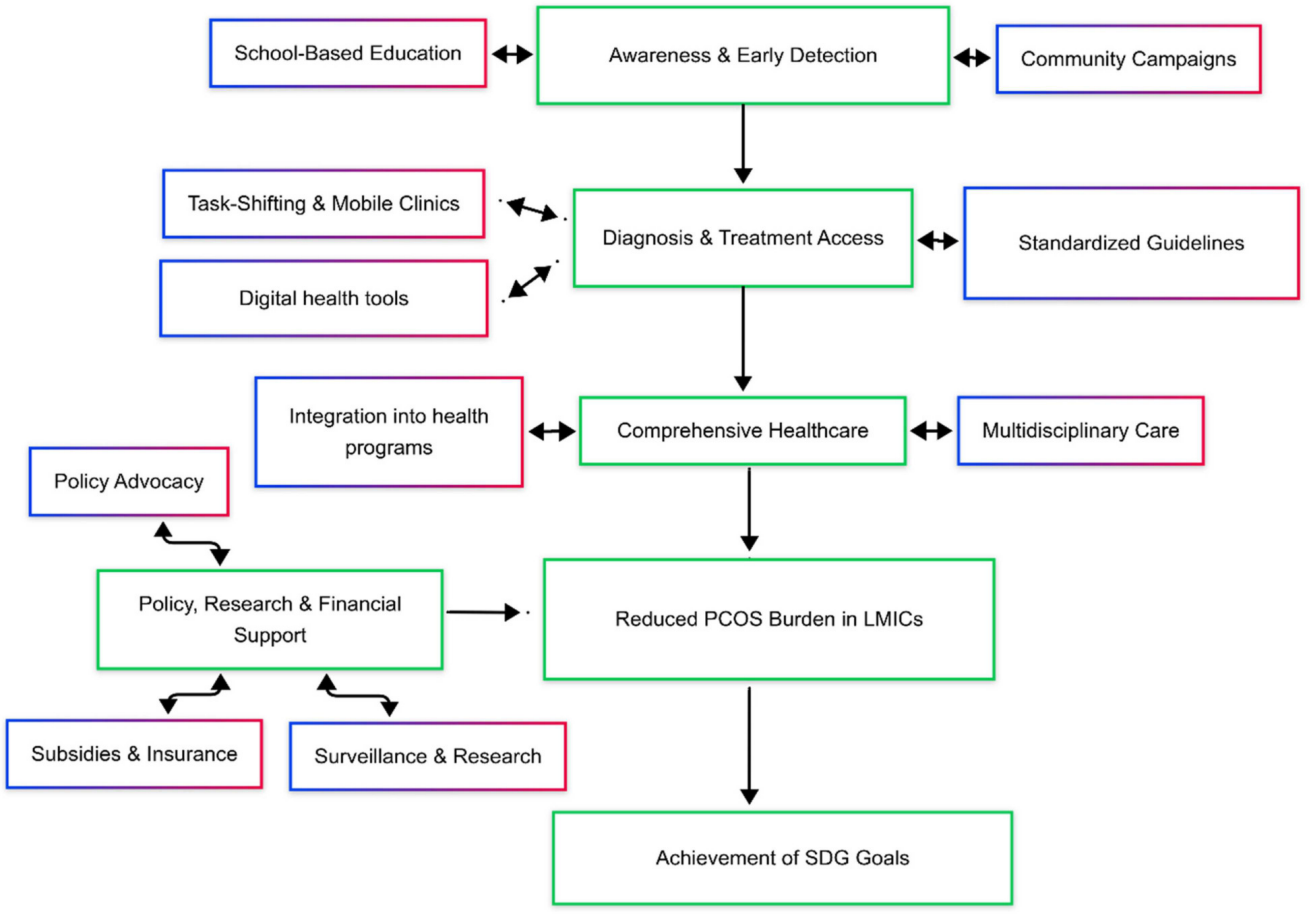
Framework for comprehensive PCOS management in LMIC.

This section discusses, public health solutions like, prevention and early intervention approaches for PCOS, models of cost-effective care and policy changes (Figure 2).

### 3.1 Prevention and early intervention in PCOS

**Figure 3:**
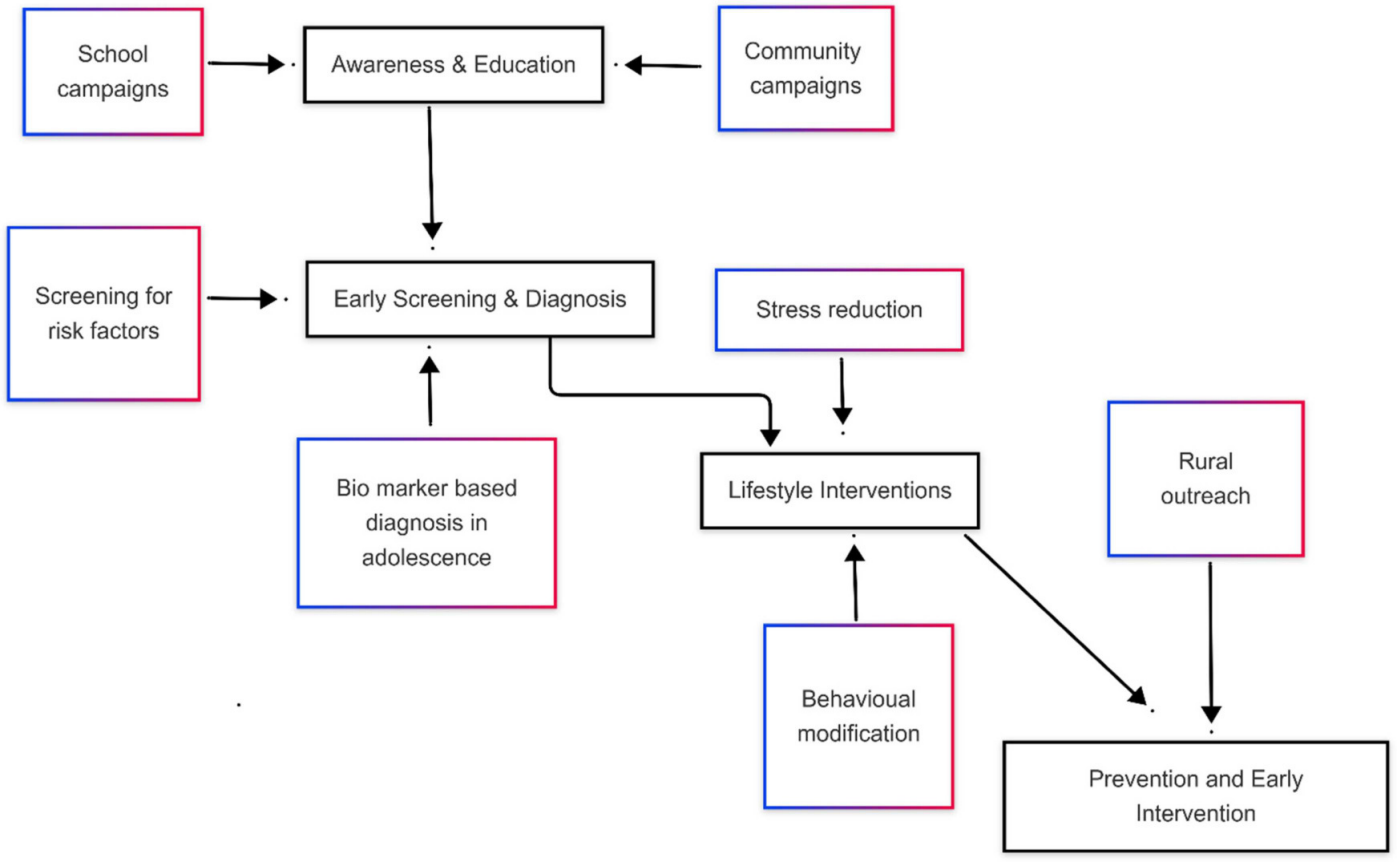
Prevention and early intervention framework for PCOS care in LMIC.

Promoting lifestyle modifications, implementing early diagnosis and treatment are effective strategies contribute holistic care for those diagnosed with PCOS, consequently healthcare costs can be reduced (51, 52). Early intervention in PCOS management is crucial to the mitigation of type 2 diabetes, cardiovascular disease, and infertility (53). Furthermore, achieving these goals will require joint action by governments, healthcare providers, educators, and community leaders (54). The screening for risk factors will facilitate lifestyle interventions in time, which are more effective when started soon (55). All three mode of prevention the primary, secondary and tertiary plan an important role in reducing the burden of PCOS and its complications in the community (Figure 3).

#### 3.1.1 Early screening and diagnosis

The timely detection of PCOS is important (56). The period of adolescence is the golden time when it comes to the detection of the earliest symptoms of PCOS that are mostly related to menstrual disturbance, acne, and facial hair (55, 57). Another intervention that can assist in reaching more people in the community is through the engagement of local leaders and organizations. The roll out of mobile health units with primary diagnostic tools means the screening service can be brought to remote areas and the barriers of geography and finance can be overcome. Biomarkers such as AMH, INH-A, and INSL3 are found to be useful in some studies in the early diagnosis of PCOS in adolescents, there is currently no consensus on their diagnostic thresholds (58).

#### 3.1.2 School-based preventive approaches

Educating adolescent girls about menstrual health and the risk factors of PCOS is a cost-effective strategy for recognition of risk factors related to PCOS and encouraging healthy behaviours (59). School-based programs can include workshops and sessions that normalize discussions about menstrual health can help dispel myths and reduce stigma (25). Educating young women can emphasize the importance of a balanced diet, regular exercise, and stress management. Incorporating these evidence-based practices into school curricula ensures that students receive consistent support mitigate lifestyle diseases like PCOS (12). Parents and teachers often play a crucial role in supporting the children, hence sensitizing them is also important for the sustainability of primary prevention strategies (35).

#### 3.1.3 Early interventions

Lifestyle interventions like exercise and dietary modification, is an important cornerstone of PCOS prevention and management (55, 56). Evidence showcases the important role of dietary changes, regular physical activity, and stress management in alleviating symptoms and reducing the risk of complications (60). Nutritional interventions are important in managing insulin resistance, a common feature of PCOS. Diets rich in whole grains, lean proteins, and vegetables, along with reduced intake of processed foods and sugary beverages, have been shown to improve metabolic and reproductive outcomes (61). However, implementing such dietary changes can be challenging in low-resource settings due to food insecurity and limited access to nutritional counselling (62).

Regular exercise has been proven to improve insulin sensitivity, reduce androgen levels, and promote weight loss in women with PCOS. Even moderate-intensity activities, such as brisk walking or yoga, can yield significant benefits (63). Community-based initiatives that create safe spaces for exercise and promote group activities can help overcome barriers to physical activity in rural areas (24).

Chronic stress exacerbates PCOS symptoms by disrupting hormonal balance. Stress-reduction techniques, such as mindfulness, meditation, and cognitive-behavioural therapy, are effective in improving mental health and overall quality of life (64). Integrating these practices into community programs can make stress management accessible to women in low-resource settings.

In low-resource settings, dietary change programs can be supported by school-based initiatives, women-led self-help groups, and nutritional counselling included in primary healthcare systems. In India, similar models have shown success in tackling child and maternal under nutrition (65–67).

### 3.2 Cost-effective models of care for tackling PCOS

#### 3.2.1 Task-shifting models

Task-shifting models represent a strategy of redistributing responsibilities from specialists to trained nurses and primary care physicians (68). These models can play an important role in improving accessibility to PCOS management (43). Such methods have the potential to reduce the burden placed on specialists by giving primary care providers the knowledge they need to diagnose and manage PCOS effectively (69). Training courses aimed at nurses and primary care physicians consist of identifying main symptoms including the irregular menstruation, hyperandrogenism, and polycystic ovarian morphology, as well as offering lifestyle interventions and pharmacological treatments (70) (Table 2 and Figure 3).

**Table 2:** Cost effective models of care in PCOS.

Intervention	Description	Evidence and references
Task-shifting models	Redistributing responsibilities from specialists to trained nurses, primary care physicians, and community health workers (CHWs).	Successful in South Africa (NCD care), HIV care models, CHW-led healthcare in India (71).
	Includes early identification, lifestyle interventions, and follow-up for metabolic disorders.	Empowering CHWs with portable diagnostic tools improves adherence and follow-up (72, 73)
Digital health in primary care	Telemedicine provides virtual consultations, reducing travel costs and improving access for rural populations.	Mobile health applications improve adherence to lifestyle changes and medication digital risk profiling (74).
	Enables primary care physicians to consult specialists remotely for diagnosis and treatment adjustments.	Digital health use in cardiovascular risk profiling and personalized care in New Zealand (9, 75).
Community mobilization	Mass campaigns raise awareness about PCOS symptoms and promote early diagnosis and health-seeking behavior.	Campaigns leveraging radio, TV, social media, and events reduce stigma and encourage preventive care (76).
	Collaborative programs with schools, workplaces, and community groups enhance early recognition and support systems.	Workshops for adolescents and male engagement help foster supportive (77).

Adding task-shifting models can also result in earlier diagnosis and management, thus preventing such long-term complications as infertility, type 2 diabetes, and cardiovascular disease (69). Of note, care to guarantee geographical and economic access is also obtained by these models, and thereby it enables PCOS management to be brought within reach of women in rural and the most underserved regions (50). The implementation plan consists of rigorous training programs, regular supervision, and the embedding of evidence-based guidelines into primary care settings (70).

Successful case studies of utilization of task shifting models for non-communicable disease care has been reported in South Africa. Where screening for hypertension which was traditionally done by physician were reallocated to physician assistants (78). Similar examples have also reported in HIV care, where nurses took over the roles traditionally done by physicians (79).

Community health workers (CHWs) have long been instrumental in delivering primary healthcare services in underserved regions (80). The potential of CHWs Like CHOs, MPWs, ANM to bridge gaps in PCOS care by leveraging their trusted position within communities is promising and has been proven effective in other health programs in India (81, 82)Empowering CHWs with portable diagnostic tools, such as glucometers and blood pressure monitors, they can assist in the early identification, health education and follow up of associated metabolic disorders, thereby improving treatment adherence (78, 49). Such initiatives require comprehensive training modules, adequate remuneration, and support systems to maintain CHW motivation and effectiveness (79).

The cost-effectiveness of these three approaches, task-shifting to primary care physicians, structured training for nurses, and CHW-led interventions, has been clearly shown in LMIC health systems. In Uganda, training nurses to conduct cardiovascular and metabolic risk screening cut per-patient costs by 30% compared to physician-led services, without affecting diagnostic accuracy (83). In Tanzania, shifting PMTCT (Prevention of Mother-to-Child Transmission) tasks to nurses and midwives reduced nurse-to-patient time by 25% and brought notable cost savings for the health system (84). CHW-based hypertension management programs in Cameroon demonstrated that giving CHWs portable diagnostic devices and referral protocols was both cost-effective and sustainable. This approach lowered uncontrolled hypertension prevalence at a cost of less than USD 3 per patient each year (85). These findings highlight that when such models include structured supervision, evidence-based protocols, and performance-linked incentives, they can provide high-quality, affordable PCOS-related services in resource-limited settings.

Simple body measurements such as waist-to-hip ratio (WHR) and waist-to-height ratio (WHtR) strongly relate to insulin resistance and metabolic syndrome in PCOS. They offer a practical, low-cost option for early risk assessment in field situations (86).

#### 3.2.2 Digital health in primary care

Advancement in technology even brought telemedicine which is a game changer in the way we should approach PCOS care for those women who live in the countryside and accessible areas of the country (87). Telemedicine is the provider's vehicle to the patients through virtual check-ups, which in return decrease the need for travel and lessen the costs (88).

Moreover, telemedicine assists patients’ interaction through educating materials and guiding on the modification of their lifestyles according to their needs (52). Telemedicine would enrich an already established healthcare infrastructure by combining a primary care provider with specialists. For instance, general practitioners can communicate with endocrinologists or gynaecologists virtually for confirmations of diagnoses, or treatments refinements (88). The main advantage of telemedicine is its capability carter to a larger population of the people without a need to correspondingly to increase the workforce. But problems such as digital literacy, internet connection, and data protection concerns must be resolved in the desire to realize equality of access and usage (53).

Studies in Asia has shown that the use of mobile application in helping improve patient adherence to lifestyle intervention in NCD care. A study in New Zealand has showcased the use of digital health in risk profiling in cardiovascular illness, using real time data, which helps make personalised treatment strategies and improve health outcomes (89). A study has found that use of remainders telephone messages improve adherence to medications (90).

New Zealand's CONNECT trial showcased the viability of app-based follow-ups and health coaching for chronic conditions, which includes work with metabolic disorders (75). In the same vein, India's e-Sanjeevani platform has expanded the delivery of digital health services to rural primary health centres (PHCs). Nevertheless, digital literacy and infrastructural gaps need to be bridged (87). In Pakistan, a mobile health intervention focused on reproductive disorders improved symptom tracking and adherence to follow-up (74). Brazil's “Telessaúde” program, designed for remote consultation and education, demonstrated high user satisfaction among women with hormonal disorders, including PCOS (88). India's e-Sanjeevani telemedicine platform has successfully delivered NCD and reproductive health consultations across rural and urban areas, including states with limited specialist availability (91, 92). We have also acknowledged critical barriers such as low digital literacy, inconsistent internet access, and privacy concerns, which could hinder PCOS-specific digital health interventions in LMICs unless addressed through supportive infrastructure and training (93).

#### 3.2.3 Community mobilization

Raising awareness about PCOS through mass campaigns is essential to reduce stigma, encourage early diagnosis, and promote health-seeking behaviour (94). It is also important to use culturally sensitive materials and local languages to engage diverse populations effectively. Awareness campaigns can address misconceptions about PCOS, highlight its symptoms, and educate women about the benefits of timely intervention (8).

Such campaigns can leverage various media, including radio, television, social media, and community events, to disseminate information (61). Collaborations with schools, workplaces, and community organizations can amplify the reach and impact of these efforts. For example, organizing workshops and seminars targeting adolescent girls and young women can foster early recognition of symptoms and encourage preventive measures. Additionally, involving male family members in awareness programs can help build supportive environments for women seeking care (62).

Mass campaigns also play a pivotal role in advocating for policy changes and increasing public investment in PCOS care. By generating widespread awareness and demand for services, these initiatives can drive the expansion of healthcare infrastructure and resources dedicated to PCOS management (1). However, sustained funding and strategic planning are critical to ensuring the long-term success of awareness campaigns (63).

### 3.3 Public health policy needs for PCOS management

#### 3.3.1 Integration into reproductive health programs

Embedding PCOS diagnosis and management into existing reproductive and maternal health programs can optimize resource utilization and improve accessibility. Training and incentivising existing public health human resources like PHC medical officers, Accredited Social Health Activists (ASHAs) and Auxiliary Nurse Midwives (ANMs) to screen for PCOS symptoms and provide basic counselling can be a cost-effective solution (95, 96). This approach leverages existing healthcare infrastructure, particularly in rural areas, to identify women at risk and provide timely interventions. US veteran affairs have incorporated PCOS into their program, they have trained their primary health care professionals to diagnose and treat minor manifestations of PCOS. Addressing PCOS requires collaborative efforts involving government agencies, non-governmental organizations (NGOs), and private healthcare providers (64). Multi-sectoral partnerships enhance the reach and efficacy of PCOS management programs (97).

Integrating chronic disease management into reproductive health services has shown clear success in low- and middle-income countries and sets a strong example for managing PCOS. In Tanzania, including hypertension and diabetes screening in HIV and antenatal clinics cut patient travel time by 40% and increased detection rates for non-communicable diseases without needing additional staff (98). In Bangladesh, reproductive health workers trained to provide combined maternal health and non-communicable disease services saw a 25% rise in early diagnosis of metabolic disorders among women of reproductive age (99).

#### 3.3.2 National guidelines for PCOS management

The establishment of standardized national guidelines tailored to low-resource settings is essential for consistent and effective PCOS management (100). A simplified diagnostic criteria and accessible treatment protocols, including lifestyle interventions and affordable pharmacological options. Although international guidelines are available, national guidelines are realistic, culturally sensitive and should prioritise the unique challenges faced by underserved populations, such as limited access to healthcare and financial constraints (100) Australia's National Health and Medical Research Council, has developed a guideline for comprehensive management of PCOS (101).

Nationally adapted guidelines for chronic disease care in low- and middle-income countries show that standardization can improve consistency and quality in settings with limited resources. In South Africa, the Primary Care 101 guideline incorporates algorithms for non-communicable diseases into primary health care practice (102). This allows nurses to start evidence-based management with little input from specialists, resulting in better adherence to clinical protocols and fewer inappropriate referrals. In India, the NPCDCS program standardizes the management of hypertension and diabetes across states with simple flowcharts and training modules for non-specialist providers (103). Adapting PCOS guidelines at the country level, with straightforward diagnostic criteria and culturally relevant lifestyle recommendations, would increase feasibility in resource-limited environments.

#### 3.3.3 Subsidized healthcare services

Subsidizing diagnostic tests, medications, awareness generation through mass campaigns and lifestyle counselling can alleviate the financial burden of PCOS management for low-income populations (56, 104, 105). The policy interventions that make essential healthcare services more affordable and accessible. Providing incentives to private healthcare providers to offer low-cost services can further expand the availability of care (50). Subsidized programs can ensure that economic constraints do not hinder women from seeking timely diagnosis and treatment, ultimately improving health outcomes at the population level (57). In countries like Canada, the provincial health insurance plans cover consultations, diagnostic tests, and treatments of PCOS management.

In Kenya, government-funded non-communicable disease clinics offer free screening for diabetes and hypertension, reducing out-of-pocket costs and improving follow-up (99). In Sri Lanka, essential medicines for non-communicable diseases are fully subsidized through the public health system, with free metabolic screening available in government hospitals (15). Applying this model to PCOS could include subsidizing hormone tests, ultrasound imaging, and metformin, along with community-based lifestyle counselling, to reduce financial barriers for low-income women.

#### 3.3.4 Multi-disciplinary care

PCOS being a muti faceted causes manifestations like the reproductive, metabolic, dermatological and psychological. These might require care from various specialists like obstetricians, endocrinologists, dermatologists and psychiatrist for comprehensive care. Centres like University of California, San Francisco, National institute for research in reproductive health conduct multi-disciplinary clinics to treat PCOS cases (16, 64). There should be a policy to develop infrastructure for multi-disciplinary clinics all tertiary care hospitals across LMICs.

In South Africa, multidisciplinary NCD clinics bring together reproductive, metabolic, and mental health services in one place. This setup lowers patient drop-out rates and helps people stick to lifestyle changes (13). In Brazil, the Family Health Strategy connects community health workers with specialists. This approach ensures ongoing support and early treatment for women with reproductive and metabolic issues (14). These models show that even in areas with limited resources, teamwork among different specialties, backed by referral networks and shared care plans, can lead to better results for PCOS while making the most of available specialist resources.

### 3.4 Research priorities for comprehensive management of PCOS

#### 3.4.1 Epidemiological studies

Large-scale epidemiological studies are crucial to understanding the true prevalence and phenotypic variations of PCOS in India (106). There is a need for comprehensive research that includes both rural and urban populations, providing insights into the disease burden and its socio-demographic correlates. Such studies can inform public health policies and resource allocation, ensuring that interventions are tailored to the needs of diverse populations (3) (Table 3).

**Table 3:** Research priorities for comprehensive management of PCOS.

Research area	Description	Evidence and references
Epidemiological studies	Large-scale studies to find the prevalence and phenotypic variations of PCOS in diverse populations.	(2)
Cost-effective diagnostic tools	Developing simplified ultrasound protocols and low-cost biomarker tests to improve accessibility.	(107)
Role of community, culture, and economics	Community-based lifestyle programs leveraging local resources for sustainable interventions.	(108)
Implementation research	Evaluating training programs for primary healthcare workers and integrating digital health solutions.	(8)
Screening and surveillance	Developing and validating surveillance tools for early PCOS detection and monitoring high-risk groups.	(109)

#### 3.4.2 Cost-effective diagnostic tools development

Developing low-cost diagnostic tools, such as simplified ultrasound protocols or biomarker-based tests, can significantly improve accessibility in resource-constrained settings (110). Affordable one prick diagnostic tools are particularly important in rural areas, where access to advanced healthcare facilities is often limited (17) (Table 3). More affordable markers of insulin resistance and hepatic steatosis in PCOS, such as the triglyceride-glucose index, TG/HDL ratio, HOMA-IR, and ALT/AST ratio, have been validated in numerous LMIC populations (107).

#### 3.4.3 Role of community, culture and economics

Pilot studies evaluating community-based lifestyle intervention programs can provide evidence for scalable solutions to PCOS management (55). Community, school and workplace-based approaches are effective for helping sustain dietary modifications, exercise regimens, and mental health counselling in improving metabolic and reproductive outcomes. Community-based interventions leverage local resources and social networks, making them cost-effective and culturally appropriate (59).

Research exploring the cultural and socioeconomic determinants of PCOS can inform the development of targeted interventions (58). The importance of understanding barriers to healthcare access and adherence in different communities. Addressing these factors can enhance the acceptability and effectiveness of public health programs, ensuring that they meet the unique needs of diverse populations (51).

#### 3.4.4 Implementation research

Implementation research is critical to integrating PCOS management into existing healthcare systems. There is a need to evaluate training programs for primary healthcare providers and community health workers and assessing effectiveness of improving delivery of these using CHWs lead community-based interventions (54).

The role of digital health tools, such as mobile apps for symptom tracking and telemedicine for remote consultations, should be explored as cost-effective alternatives for PCOS management (68). The digital platforms can enhance access to care, particularly in remote and underserved areas. These tools also promote patient engagement and self-management, empowering women to take an active role in their health (64).

#### 3.4.5 Screening and surveillance

Screening and surveillance strategies and tools can help in early detection of PCOS risk factors and symptoms among adolescents and young women, studies can be made on developing and validating such surveillance tools and algorithms (111, 112). Sentinel surveillance sites can be developed at facilities where people with high PCOS risk show up regularly to monitor the trends of the risks, disease and its complications among the target group.

## 4 Conclusion

To improve PCOS management in low resource setting where there is inadequate availability of trained health professionals, certain components of it can be merged into primary healthcare systems to improve early identification and comprehensive care. Task shifting models that have proven effective for other programs including diabetes mellitus control program, which involves training community health workers, such as ASHAs and ANMs, can increase awareness reduce stigma and promoting early detection. Embedding PCOS management into reproductive and maternal health programs can utilize existing infrastructure for better resource utilization. Simultaneously, establishing national guidelines tailored to resource-constrained settings ensures equitable and consistent care delivery.

While lowcost options like metformin are available, care should be taken to prescribe those cost-efficient medications though primary care centres, and subsidized tertiary care can ensure access to care, and catastrophic expenditure for low-income populations. Research into cost-effective diagnostic tools, scalable lifestyle interventions, and digital health solutions is vital for addressing gaps in PCOS management. A coordinated effort involving government, NGOs, and private stakeholders is crucial for comprehensive management of PCOS to achieve SDG by 2030. By prioritizing prevention, affordability, and accessibility, public health policies can reduce the burden of PCOS and help mitigate the burden of PCOS and its complications.
